# Evidence of Adaptation to Recent Changes in Atmospheric CO_2_ in Four Weedy Species

**DOI:** 10.3390/plants7010012

**Published:** 2018-02-19

**Authors:** James Bunce

**Affiliations:** Crop Systems and Global Change Laboratory, USDA-ARS, Beltsville, MD 20704, USA; buncejames49@gmail.com; Tel.: +1-410-451-7343

**Keywords:** CO_2_, adaptation, photosynthesis, growth, weeds, carboxylation efficiency

## Abstract

Seeds of three C_3_ and one C_4_ annual weedy species were collected from agricultural fields in Beltsville, Maryland in 1966 and 2006, when atmospheric CO_2_ concentrations averaged about 320 and 380 mol mol^−1^, respectively. Plants from each collection year were grown over a range of CO_2_ concentrations to test for adaptation of these weedy species to recent changes in atmospheric CO_2_. In all three of the C_3_ species, the increase in CO_2_ concentration from 320 mol mol^−1^ to 380 mol mol^−1^ increased total dry mass at 24 days in plants from seeds collected in 2006, but not in plants from seeds collected in 1966. Shoot and seed dry mass at maturity was greater at the higher growth CO_2_ in plants collected in 2006 than in 1966 in two of the species. Down-regulation of photosynthetic carboxylation capacity during growth at high CO_2_ was less in the newer seed lots than in the older in two of the species. Overall, the results indicate that adaptation to recent changes in atmospheric CO_2_ has occurred in some of these weedy species.

## 1. Introduction

Gene frequencies in genetically diverse populations respond to environmental change, and unidirectional environment change should lead to shifts in gene frequencies. Rising atmospheric carbon dioxide concentration is such a unidirectional change. Tests of adaptation to imposed elevated CO_2_ concentrations have been rather inconclusive [1,2], but that could be because the elevated CO_2_ concentrations tested may be stressful in some ways, as evidenced, for example, by photosynthetic down-regulation [3]. The concentration of carbon dioxide in the atmosphere has been gradually increasing since the beginning of the Industrial Revolution in Europe, from a concentration of about 280 mol mol^−1^ [4]. Because C_3_ photosynthesis usually remains limited by CO_2_ availability, even at the current concentration of about 400 mol mol^−1^, the past increase has represented an increase in a growth-limiting resource for many plants [3]. Bunce [5] found that four annual weedy C_3_ species were better adapted to the current atmospheric CO_2_ concentration in several aspects, including photosynthetic carboxylation capacity, than they were to the pre-industrial concentration. This suggested that adaptation to recent changes in the atmospheric CO_2_ concentration had probably occurred in these species. Comparisons of growth and photosynthetic characteristics of older and newer crop cultivars have had variable results, sometimes with higher rates in newer cultivars [6,7], but no differences in other cases [8,9]. However, in all of those studies plants were only grown at the current ambient CO_2_, not at the prior concentration, so possible adaptation to the increase in CO_2_ was not evaluated. The few tests in crop species of whether growth at projected higher future CO_2_ concentrations selected for plants with higher growth rate at elevated CO_2_ have sometimes, but not always, found higher growth rates [10,11]. Some studies have found that exposure of populations of non-cultivated plants to elevated CO_2_ resulted in adaptation to the elevated concentration, as shown by more rapid growth rates and/or increased reproduction [12,13,14,15,16,17], but there are other cases in which this did not occur [18,19,20]. In this study, I compared the response of both growth rates and photosynthetic properties to growth CO_2_ concentration in seeds of four annual weedy species collected in the same location about 40 years apart in order to more directly test for evidence of adaptation to changes in CO_2_ concentration in the recent past in these species. The primary hypothesis was that the growth of seedlings from newer seed lots would show greater increase in growth from 320 mol mol^−1^ to 380 mol mol^−1^ CO_2_ concentration than plants would from the older seed lots. A secondary hypothesis was that plants from more recent seed lots would have less down-regulation of photosynthesis when grown at elevated CO_2_.

## 2. Results

### 2.1. Seedling Growth

In all three of the C_3_ species, the increase in CO_2_ concentration from 320 mol mol^−1^ to 380 mol mol^−1^ increased the total dry mass at 24 days after planting in plants grown from seeds collected in 2006, but not in plants grown from seeds collected in 1966 (Table 1). Mean leaf area ratios for days 20 and 24 did not differ between seed lots, or with growth CO_2_ in *A. theophrasti* or *C. album*. In *D. stramonium* and *A. hybridus*, mean leaf area ratios were decreased at the higher growth CO_2_. Relative growth rate from day 20 to 24 differed between 320 mol mol^−1^ and 380 mol mol^−1^ only in the cases of *C. album* and *D. stramonium*, for the newer seed lots (Table 1).

### 2.2. Dry Mass at Maturity

In *C. album*, flowering did not occur in most of the individual plants from either era by the time of seed maturity of the other species, so for this species, only shoot biomass at 57 days after planting was obtained. The newer seed lot of *C. album* produced more shoot biomass, and more shoot biomass was produced at the higher than at the lower CO_2_ for seed lots from both eras (Table 2). *A. theophrasti* had the same response pattern as in *C. album* for both total and seed biomass, with increases with growth CO_2_ concentration, and higher mass for the newer seed lot at both CO_2_ concentrations. In *D. stramonium*, total shoot and seed dry mass were increased by the growth CO_2_ for both seed lots, but no differences occurred between seed lots. In *A. hybridus* total shoot and seed dry mass at maturity did not differ between 320 mol mol^−1^ and 380 mol mol^−1^ from either seed collection time (Table 2). Flowering occurred 6 days earlier (day 27 vs. day 33 after planting) in the newer seed lot in this species, which limited the final biomass accumulated in this determinate species.

### 2.3. Photosynthesis

**Figure 1:**
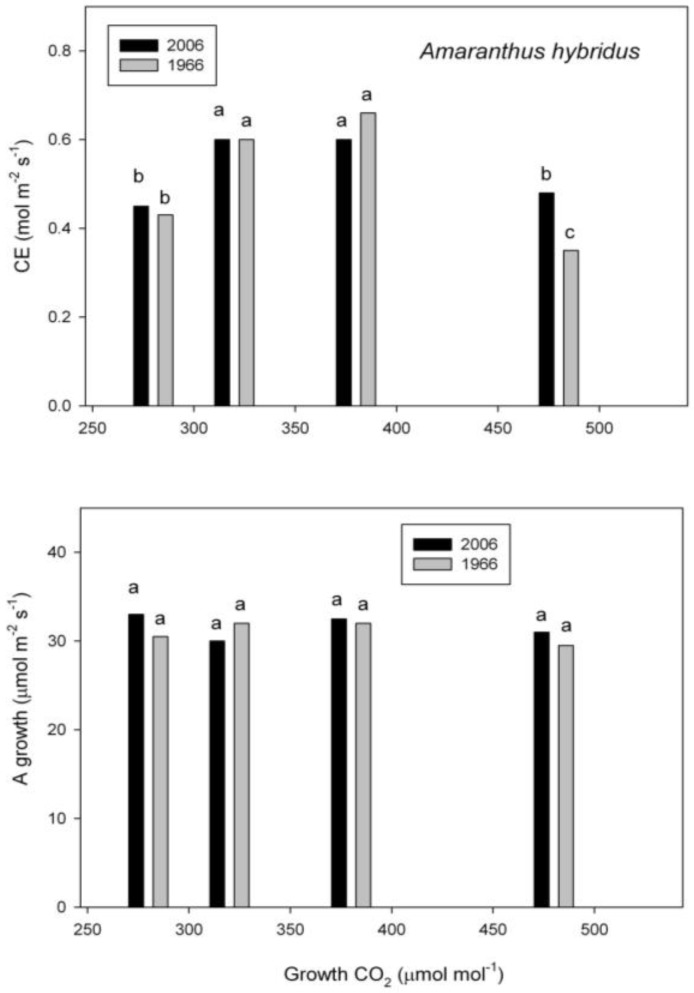
Carboxylation efficiency (CE) and assimilation rate (A) under the growth conditions in *Amaranthus hybridus* grown at four CO_2_ concentrations from seeds collected in 1966 and 2006. Different letters indicate significant differences, based on analysis of variance.

In the C_4_ species, *A. hybridus*, the carboxylation efficiency of PEPcase was reduced by growth at the lowest and highest CO_2_ concentrations (Figure 1). The reduction at the highest-growth CO_2_ in the plants from 1966 was larger than that of plants from 2006 (Figure 1). Despite differences in carboxylation efficiency, rates of photosynthesis under the growth conditions were the same for all growth CO_2_ concentrations in this species, and did not differ between seed lots, even at the highest-growth CO_2_ (Figure 1).

**Figure 2:**
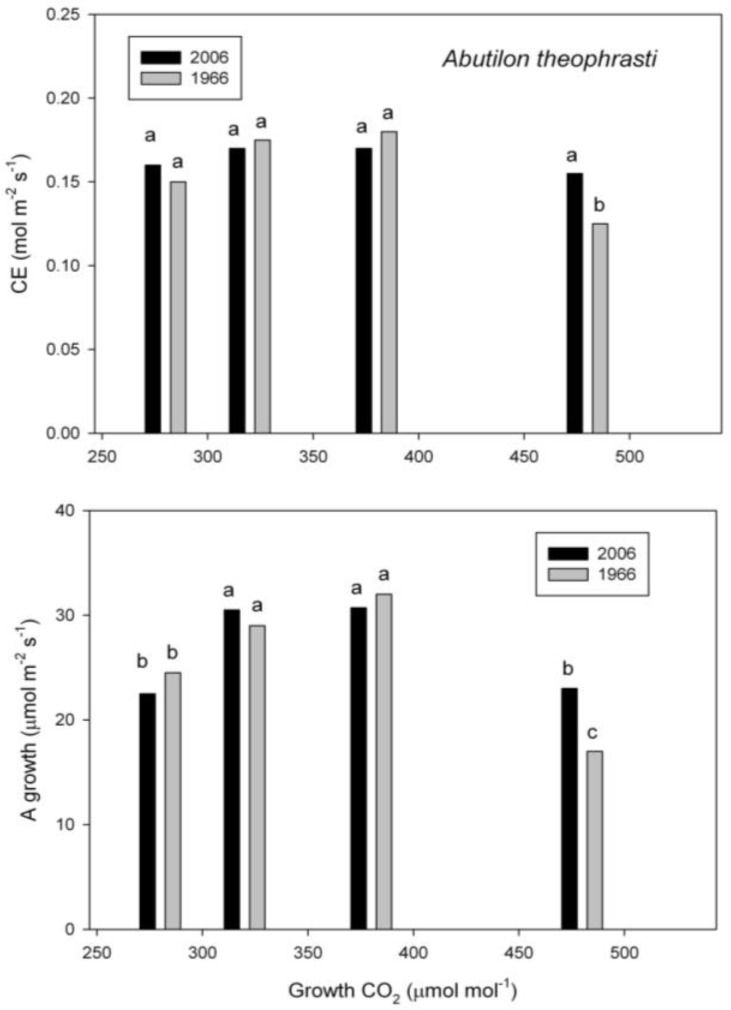
Carboxylation efficiency (CE) and assimilation rate (A) under the growth conditions in *Abutilon theophrasti* grown at four CO_2_ concentrations from seeds collected in 1966 and 2006. Different letters indicate significant differences, based on analysis of variance.

In *A. theophrasti*, the carboxylation efficiency of Rubsico was also reduced at the highest-growth CO_2_, but only in the plants from the 1960s (Figure 2). At the higher-growth CO_2_, photosynthesis under the growth conditions was also lower in the older seed lot than in the newer. Photosynthesis under the growth conditions was also lower at the lowest growth CO_2_ than at the intermediate CO_2_ concentrations (Figure 2).

**Figure 3:**
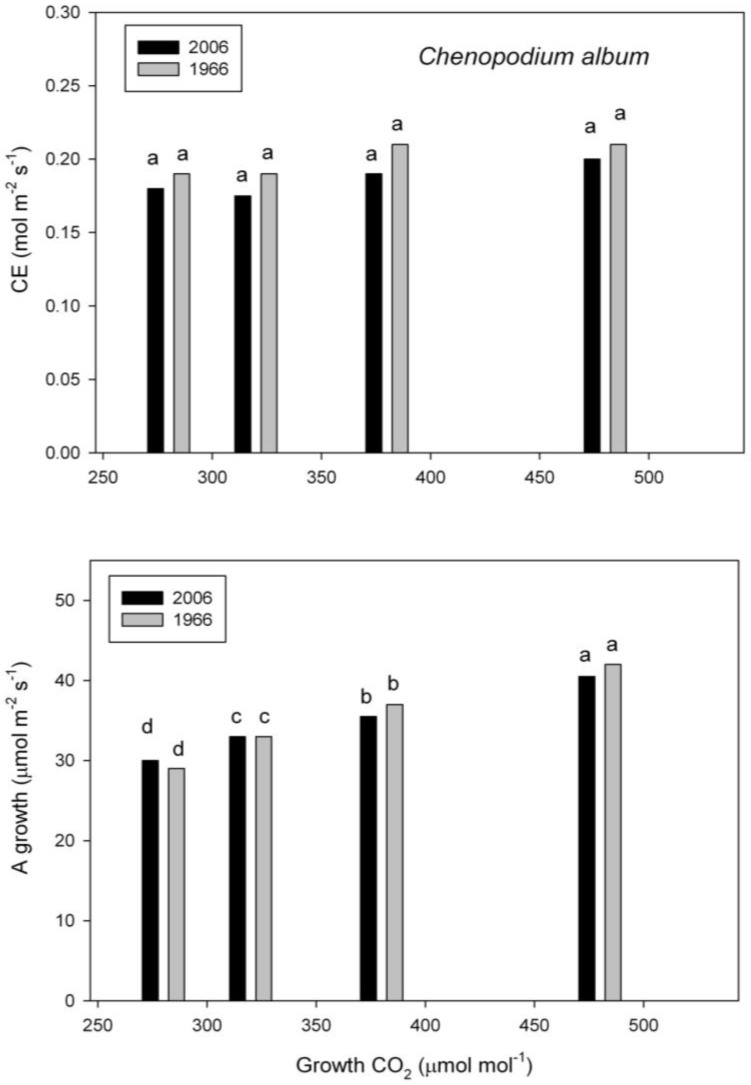
Carboxylation efficiency (CE) and assimilation rate (A) under the growth conditions in *Chenopodium album* grown at four CO_2_ concentrations from seeds collected in 1966 and 2006. Different letters indicate significant differences, based on analysis of variance.

In *C. album*, carboxylation efficiency of Rubisco was unaffected by the growth CO_2_, and never differed between old and new seed lots (Figure 3). Rates of photosynthesis under the growth conditions increased with growth CO_2_ in both seed lots, and never differed between seed lots (Figure 3).

**Figure 4:**
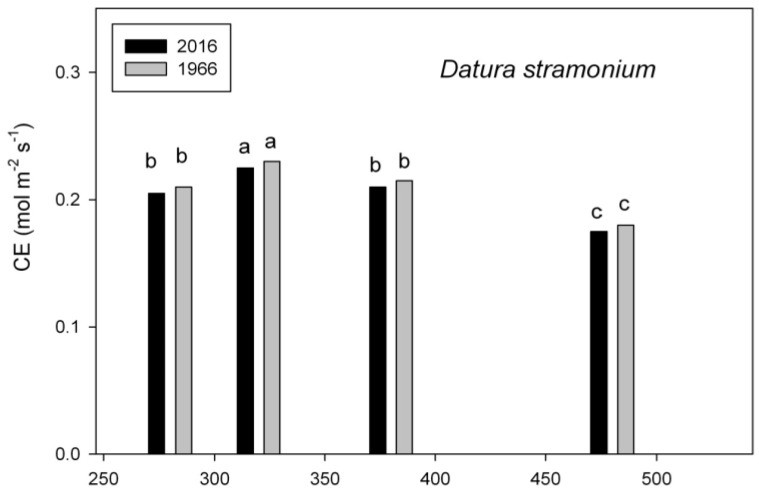

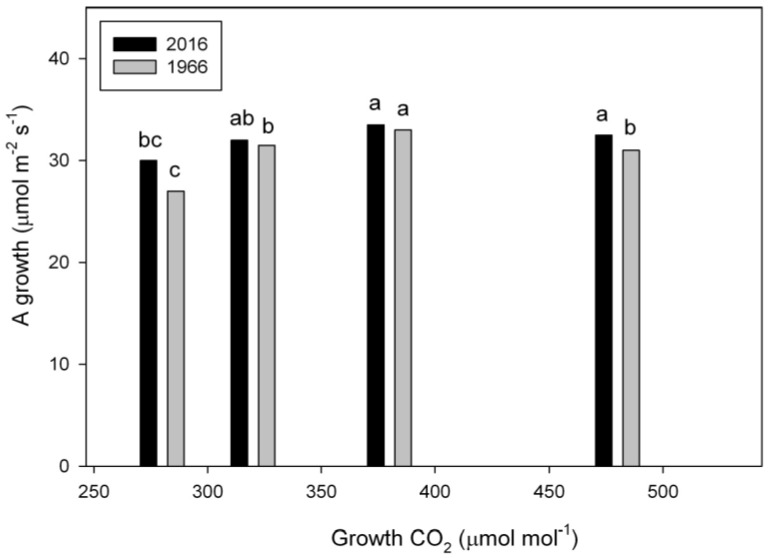
Carboxylation efficiency (CE) and assimilation rate (A) under the growth conditions in *Datura stramonium* grown at four CO_2_ concentrations from seeds collected in 1966 and 2006. Different letters indicate significant differences, based on analysis of variance.

In *D. stramonium*, carboxylation efficiency of Rubisco was highest at the growth CO_2_ concentration of 320 mol mol^−1^ in both seed lots, and did not differ between old and new seed lots at any growth CO_2_ (Figure 4). Photosynthesis under the growth conditions increased slightly with increasing growth CO_2_, up to 380 mol mol^−1^ in this species (Figure 4).

## 3. Discussion

There are three results from this experiment indicating that the seeds collected 40 years apart differed in adaptation to the CO_2_ environment. One of these results was that seedlings grown from seeds collected about 1966 did not increase in biomass at 20 or 24 days after planting when grown at 380 mol mol^−1^ vs. 320 mol mol^−1^ CO_2_, whereas seedlings collected from seeds in 2006 were larger when grown at the higher CO_2_ concentration. This pattern occurred in all three of the C_3_ species. Because all of the experimental plants were grown together simultaneously in the same chambers, yet responded differently, environmental differences among chambers can be eliminated as causing the contrasting results between seed lots. The second result indicating adaptation rising atmospheric CO_2_ was the larger final seed mass and/or shoot biomass in plants from the newer than from the older seed lot when plants grown at the higher CO_2_ concentration, which occurred in two of the species studied here. Again, the lack of differentiation in *D. stramonium* grown at the same time eliminates other environmental differences as a cause of the observed differential response. The differential flowering times in the two seed lots of *A. hybridus* indicates that genetic change occurred over time in that species, but any relationship to changes in atmospheric CO_2_ is unclear, although CO_2_ effects on flowering time are well known [21]. These two results partially support our primary hypothesis of greater growth stimulation from 320 mol mol^−1^ to 380 mol mol^−1^ CO_2_ in newer than in older seed lots.

The third result indicating that adaptation to rising atmospheric CO_2_ occurred was the difference in photosynthetic acclimation to growth at elevated CO_2_ between the older and newer seed lots, which occurred in two of the species studied. Growth at an elevated CO_2_ concentration resulted in more down-regulation of photosynthesis in plants from the older seed lots, which partially supports our secondary hypothesis. While the growth CO_2_ concentration of 480 mol mol^−1^ may seem unreasonably high for a treatment, concentrations of CO_2_ in the field at Beltsville are often at least 100 mol mol^−1^ above the midday concentration for several hours in the morning, when wind speed is low [22]. Prior experiments with *C. album* also had found no evidence of down-regulation of photosynthesis during growth at elevated CO_2_ in this species [5]. For the C_4_ species *A. hybridus*, it is not surprising that photosynthetic rates under the growth conditions did not reflect the observed down-regulation of carboxylation efficiency, because rates of photosynthesis in C_4_ species are generally only limited by carboxylation efficiency during periods of soil or atmospheric water stress.

Studies comparing photosynthesis of old and new crop cultivars [6,7,8,9] have only measured photosynthesis under the current growth CO_2_ concentration, not the CO_2_ concentration at the time of cultivar release or at projected higher concentrations. The photosynthetic characteristics of the weeds studied here measured only at the mean CO_2_ concentration of 2006 did not indicate any differences between the seed lots from different years, similar to the results found in wheat [8] and one study of soybean [9].

## 4. Materials and Methods

Seeds of four annual weedy species, *Abutilon theophrasti* (Medikus), *Amaranthus hybridus* (L.), *Chenopodium album* (L.), and *Datura stramonium* (L.) were collected in 1966 and again in 2006 from agricultural fields at the Beltsville Agricultural Research Center, Beltsville, Maryland (39°02′ N, 76°94′ W, elevation 30 m). Seeds were collected from multiple individual plants of each species and pooled within species. Seeds were air dried, and then stored at about 4 °C in sealed containers. Seed germination rate was measured in 2015 by planting 20 seeds from each of four 15 cm diameter pots filled with moist vermiculite per species in a growth chamber at 26/20 °C with 14 h of light at 1000 mol m^−2^ s^−1^ and a dew point temperature of 18 °C, monitoring emergence daily. Germination rate remained high (>70%) even in the older seed lots.

### 4.1. Seedling Growth Rates

Seeds of each species from both collection periods (mid-1960s and 2006) were grown together in two controlled-environment chambers with day/night temperatures of 26/20 °C, with 14 h of light at 1000 mol m^−2^ s^−1^ photosynthetic photon flux density (PPFD) from a mixture of high-pressure sodium and metal halide lamps, and a dew point temperature of 18 °C. The chambers had a growing area of 1 m^2^, and a growing height of 1 m. The temperature, dew point temperature, and light regimes were chosen as typical of mean values for summer days in Beltsville, Maryland. The CO_2_ concentrations of chamber air of the two chambers were 320 and 380 mol mol^−1^ each ± 10 mol mol^−1^ controlled by the injection of pure CO_2_ or CO_2_-free air under the control of absolute infrared CO_2_ analyzers that sampled the chamber air continuously. The mean atmospheric CO_2_ concentration was approximately 320 mol mol^−1^ in 1966, and 380 mol mol^−1^ in 2006 [4]. Two chambers were used in all of these experiments, with CO_2_ treatments randomly assigned to chambers in sequential trials. There were three repetitions over time of each chamber CO_2_ condition, with 10 pots per seed lot in each chamber run, with seedlings thinned randomly to one plant per pot two days after emergence. Plastic pots 15 cm in diameter were filled with 1.8 liters of medium grade vermiculite, and were flushed daily with a complete nutrient solution containing 14.5 mM nitrogen. Destructive harvests were made on days 20 and 24 after planting, in which whole plant leaf area, and leaf, stem and root dry mass were determined on 5 plants per species on each date. Two harvests a few days apart were used such that growth parameters such as relative growth and leaf area ratio could be calculated. The final harvest date of 24 days after planting was chosen such that flowering had not yet begun in any seed lot for any growth condition, because flowering slows growth rates. Analysis of variance was used to test separately for each species for differences between collection eras and growth CO_2_ concentrations, using mean values for the three chamber replications in two-way analysis of variance.

### 4.2. Growth to Maturity

For determination of plant dry mass at maturity, plants were grown in chambers in which daily changes in photoperiod were automatically programmed based on the latitude of Beltsville, Maryland, and a starting date of May 30. Air temperatures, the dew point temperature, and PPFD were set as described for the seedling growth experiments. There were 2 chambers each at 320 and 380 mol mol^−1^ CO_2_ concentration. Chamber interiors were 2 m × 3 m, with an interior height of 2 m. The lamp canopy was adjustable in height. Lamp output was controlled automatically based on a sensor held just above the tops of the plants in the center of the chamber. Plants were grown in 30 cm diameter plastic pots filled with vermiculite and watered daily or twice daily with nutrient solution. There were 5 pots of each seed lot for each species in each chamber.

### 4.3. Photosynthetic Acclimation

Plants were grown with the same air temperature, dew point temperature, CO_2_ concentration, and light conditions as for the seedling growth rate experiments, in the same two chambers as were used for seedling growth rate determinations. Because no differences among seed lots in photosynthetic characteristics were obtained at the growth CO_2_ concentrations of 320 and 380 mol mol^−1^, (see results), comparisons were also made on plants were grown at 280 and at 480 mol mol^−1^. The lower concentration approximated the atmospheric concentration just before the industrial revolution in Europe, and the highest concentration is that anticipated for about 50 years in the future. Two chambers were used in all of these experiments, with CO_2_ treatments randomly assigned to chambers in sequential trials. There were three repetitions over time of each chamber CO_2_ condition, with 5 pots per seed lot in each chamber run, with seedlings thinned randomly to one plant per pot shortly after emergence. Leaf gas exchange measurements were made on recently fully expanded upper leaves, at 22 or 23 days from planting, using a CIRAS-3 portable photosynthesis system (PP-Systems, Amesbury, MA, USA). The gas exchange system controlled leaf temperature, light, CO_2_, and water vapor pressure surrounding 2.5 cm^2^ intact sections of leaves, using an open measurement system. All measurements were made with leaf temperature controlled to the daytime growth air temperature of 26 °C, and at leaf-to-air water vapor pressure differences of 1 to 1.5 kPa. Each leaf was measured under four combinations of PPFD and CO_2_ concentration: at the growth PPFD of 1000 mol m^−2^ s^−1^ at 320 and 380 mol mol^−1^ CO_2_, and at a PPFD of 2000 mol m^−2^ s^−1^ at CO_2_ concentrations of 100 and 200 mol mol^−1^. The slope of the response of photosynthesis to substomatal CO_2_ concentration from measurements at 100 to 200 mol mol^−1^ external CO_2_ measured at the high PPFD was taken to indicate photosynthetic carboxylation efficiency. In the C_3_ species, this was taken to indicate the maximum carboxylation capacity of Rubisco [23], and the in the C_4_ species, it was taken to indicate the maximum carboxylation capacity of PEPcase [24]. Leaf gas exchange measurements were made on 3 or 4 plants of each species and collection era from each chamber run. Analysis of variance was used to test differences between collection eras and growth CO_2_ concentrations for each species, using mean values for the three chamber replications in two-way analysis of variance.

## 5. Conclusions

The results presented here provide evidence that adaptation to rising atmospheric CO_2_ concentration has occurred in three of the four weed species studied. This result is consistent with several observations of rapid physiological adaptation to imposed elevated CO_2_ conditions in populations of wild species cf. [16]. We can expect weed adaptation to climate change conditions to occur alongside any improvements in crop responses to climate change.

## Figures and Tables

**Table 1:** Seedling dry mass production for seed lots of four species collected in 1966 and 2006, grown at 320 and 380 mol mol^−1^ CO_2_ concentration. Total dry mass (DM, in grams) is for plants at 24 days after planting, and leaf area ratio (LAR, in cm^2^ g^−1^) and relative growth rate (RGR, in g g^−1^ d^−1^) are means for the period of 20 to 24 days after planting. Within species, values followed by different letters were different at *p* = 0.05, based on analysis of variance.

Species	Year of Collection	Growth CO_2_	Total DM	LAR	RGR
*A. theophrasti*	1966	320	4.9a	139a	0.29a
	1966	380	5.0a	134a	0.28a
	2006	320	4.0b	140a	0.21b
	2006	380	4.9a	136a	0.20b
*C. album*	1966	320	5.2a	127a	0.29a
	1966	380	5.3a	123a	0.31a
	2006	320	3.2b	122a	0.22b
	2006	380	5.6a	120a	0.29a
*D. stramonium*	1966	320	6.7b	154a	0.36a
	1966	380	7.1b	135b	0.32b
	2006	320	7.6b	145ab	0.32b
	2006	380	9.0a	116c	0.27c
*A. hybridus*	1966	320	4.1a	178b	0.30a
	1966	380	4.4a	134c	0.33a
	2006	320	3.7a	215a	0.32a
	2006	380	3.8a	165b	0.29a

**Table 2:** Total shoot dry mass (DM) and seed dry mass at seed maturity in four species from two years of seed collection, when grown at two CO_2_ concentrations (mol mol^−1^). Within species, values followed by different letters were different at *p* = 0.05, based on analysis of variance. na indicates not available.

Species	Year of Collection	Growth CO_2_	Total Shoot DM (g)	Seed DM (g)
*A. theophrasti*	1966	320	99c	30c
	1966	380	135b	41b
	2006	320	130b	40b
	2006	380	153a	62a
*C. album*	1966	320	46c	na
	1966	380	60b	na
	2006	320	59b	na
	2006	380	71a	na
*D. stramonium*	1966	320	315ab	172b
	1966	380	350a	193a
	2006	320	289b	172b
	2006	380	351a	205a
*A. hybridus*	1966	320	186a	78a
	1966	380	191a	82a
	2006	320	90b	62b
	2006	380	102b	60b

## References

[1] Shaw R.G., Etterson J.R. (2012). Rapid climate change and the rate of adaptation: Insight from experimental quantitative genetics. New Phytol..

[2] Anderson J.T. (2015). Plant fitness in a rapidly changing world. New Phytol..

[3] Bunce J.A. (2000). Contrasting effects of carbon dioxide and irradiance on the acclimation of photosynthesis in developing leaves. Photosynthetica.

[4] ESRL Global Monitoring. https://www.esrl.noaa.gov/gmd/ccgg/trends/full.html.

[5] Bunce J.A. (2001). Are annual plants adapted to the current atmospheric concentration of carbon dioxide?. Int. J. Plant Sci..

[6] Liu G., Yang C., Xu K., Zhang Z., Li D., Wu Z., Chen Z. (2012). Development of yield and some photosynthetic characteristics during 82 years of genetic improvement of soybean genotypes in northeast China. Aust. J. Crop Sci..

[7] Luo H.H., Zhang H.L., Zhang Y.L., Zhang W.F. (2017). Evolution of characteristics related to photosynthesis, growth and yield in some old and new cotton cultivars. Photosynthetica.

[8] Sadras V.O., Lawson C., Montoro A. (2012). Photosynthetic traits in Australian wheat varieties released between 1958 and 2007. Field Crops Res..

[9] Koester R.P., Nohl B.M., Diers B.W., Ainsworth E.A. (2016). Has photosynthetic capacity increased with 80 years of soybean breeding? An examination of historical soybean cultivars. Plant Cell Environ..

[10] Frenk G., van der Linden L., Mikkelsen T.N., Briz H., Jorgensen R.B. (2013). Response to multi-generational selection under elevated [CO_2_] in two temperature regimes suggests enhanced carbon assimilation and increased reproductive output in *Brassica napus* L.. Ecol. Evol..

[11] Alemayehu F.R., Frenck G., van der Linden L., Nikkelsen T.E., Jorgensen R.B. (2014). Can barley (*Hordeum vulgare* L. s.l.) adapt to fast climate changes? A controlled selection experiment. Genet. Res. Crop Evol..

[12] Fordham M., Barnes J.D., Bettarini I., Polle A., Slee N., Raines C., Miglietta F., Raschi A. (1997). The impact of elevated CO_2_ on growth and photosynthesis in *Agrostis canina* L. ssp. *montelucci* adapted to contrasting atmospheric CO_2_ concentrations. Oecologia.

[13] Polle A., McKee I., Balschke L. (2001). Altered physiological and growth responses to elevated [CO_2_] in offspring from holm oak (*Quercus ilex* L). mother trees with lifetime exposure to naturally elevated [CO_2_]. Plant Cell Environ..

[14] Newton P.C.D., Edwards G.E., Newton P.C.D., Carran R.A., Edwards G.R., Niklaus P.A. (2007). Plant breeding for a changing environment. Agroecosystems in a Changing Climate.

[15] Nakamura I., Onoda Y., Matsushima N., Yokoyama J., Kawata M., Hikosaka K. (2011). Pheotypic and genetic differences in a perennial herb across a natural gradient of CO_2_ concentration. Oecologia.

[16] Bunce J.A. (2012). Elevated carbon dioxide alters the relative fitness of *Taraxacum officinale* genotypes. Am. J. Plant Sci..

[17] Ward J.K., Antonovics J., Thomas R.B., Strain B.R. (2000). Is atmospheric CO_2_ a selective agent on model C_3_ annuals?. Oecologia.

[18] Bazzaz F.A., Jasienski M., Thomas S.C., Wayne P. (1995). Microevolutionary responses in experimental populations of plants to CO_2_-enriched environments: Parallel results from two model systems. Proc. Natl. Acad. Sci. USA.

[19] Steinger R., Stephan A., Schmid B. (2007). Predicting adaptive evolution under elevated atmospheric CO_2_ in the perennial grass *Bromus erectus*. Glob. Chang. Biol..

[20] Wieneke S., Prati D., Barndl R., Stocklin J. (2004). Genetic variation in *Sanguisorba minor* after 6 years in situ selection under elevated CO_2_. Glob. Chang. Biol..

[21] Springer C.J., Ward J.K. (2007). Flowering time and elevated atmospheric CO_2_. New Phytol..

[22] Bunce J.A. (2014). Limitations to soybean photosynthesis at elevated carbon dioxide in free-air enrichment and open top chamber systems. Plant Sci..

[23] Farquhar G.D., von Caemmerer S., Berry J.A. (1980). A biochemical model of photosynthetic CO_2_ assimilation in leaves of C_3_ species. Planta.

[24] Massad R.-S., Tuzet A., Bethenod O. (2007). The effect of temperature on C_4_-type photosynthesis parameters. Plant Cell Environ..

